# Impact of subthalamic nucleus deep brain stimulation on voice and speech in Parkinson’s disease

**DOI:** 10.1007/s00405-026-10063-9

**Published:** 2026-03-15

**Authors:** Ozan Tuysuz, Ozan Hasimoglu, Rıdvan Basaran, Ayca Altinkaya Uslu, Selin Ustun Bezgin, Sahin Ogreden, Ozgur Yigit

**Affiliations:** 1 Department of Otorhinolaryngology, University of Health Sciences Basaksehir Cam and Sakura City Hospital, Istanbul, Turkey; 2 Department of neurosurgery, University of Health Sciences Basaksehir Cam and Sakura City Hospital, Istanbul, Turkey; 3 Department of Language and Speech Terapy, University of Health Sciences Basaksehir Cam and Sakura City Hospital, Istanbul, Turkey; 4 Department of Neurology, University of Health Sciences Basaksehir Cam and Sakura City Hospital, Istanbul, Turkey

**Keywords:** Acoustic analysis, Deep brain stimulation, Speech, Parkinson’s disease, Voice handicap index

## Abstract

**Purpose:**

Subthalamic nucleus deep brain stimulation (STN-DBS) effectively alleviates motor symptoms in Parkinson’s disease (PD), yet its impact on speech and voice remains inconsistent. This study examined acoustic parameters, perceptual features, and speech intelligibility using a large, methodologically standardized cohort.

**Methods:**

A total of 105 PD patients (55 females, 50 males; mean age ≈ 57 years) undergoing STN-DBS were evaluated. All received posterolateral electrode placement, bilateral stimulation, and a postoperative 30–35% reduction in levodopa. Acoustic measures fundamental frequency (F0), jitter, shimmer, harmonic-to-noise ratio (HNR), and maximum phonation time (MPT) were obtained one day preoperatively and at three months postoperatively. Perceptual ratings (GRBAS), Voice Handicap Index-10 (VHI-10), and speech intelligibility (percentage of correctly produced words) were assessed concurrently.

**Results:**

Motor symptoms improved in the medication-on state (19.4 → 13.6). Acoustic analysis showed no significant postoperative change in F0, shimmer, HNR, or MPT; however, jitter improved significantly in both sexes. On the GRBAS scale, all parameters improved in females, whereas grade, roughness, and asthenia improved in males. VHI-10 scores improved overall (22.7 → 21.8; *p* = 0.031), driven primarily by females (27.5 → 25.9; *p* = 0.004). Speech intelligibility increased in the full cohort (70.1% → 71.7%; *p* = 0.01) and reached significance in females but not males. Higher age and greater motor severity correlated with reduced intelligibility, while sex, PD subtype, and stimulation parameters showed no associations.

**Conclusion:**

STN-DBS yields short-term motor improvement and produces parameter-specific speech effects. Utilizing a highly standardized “pure” cohort enhances interpretability and suggests that, under controlled conditions, STN-DBS can exert a beneficial influence on speech performance despite its multifactorial impact.

## Introduction

Parkinson’s disease (PD) is a progressive neurodegenerative disorder primarily characterized by tremor, bradykinesia, rigidity and postural instability. Levodopa remains the cornerstone pharmacological treatment for motor symptoms; however, long-term use may lead to reduced responsiveness and motor fluctuations. As a result, deep brain stimulation (DBS) has gained prominence as a surgical alternative or adjunct in advanced PD management, with numerous studies demonstrating its efficacy in improving motor performance and reducing dopaminergic medication requirements [1, 2].

Among DBS targets, the subthalamic nucleus (STN) is the most widely preferred. Although STN-DBS yields notable motor benefits, its effects on speech and voice remain controversial. Approximately 92.5% of patients with PD experience speech and communication difficulties, most commonly hypophonia and hypokinetic dysarthria, characterized by low vocal intensity, monotonous pitch, articulatory imprecision and impaired fluency [3, 4]. These deficits may arise not only from motor dysfunction but also from emotional and psychosocial factors associated with disease burden [5, 6].

Previous studies have reported that DBS may worsen vocal quality and speech intelligibility [7–10], while others have demonstrated improvements in selected parameters following stimulation [11, 12]. Thus, the vocal outcomes of STN-DBS remain a topic of debate, likely influenced by variability in patient selection, methodological design and acoustic measurement approaches across studies. While some evidence suggests enhanced phonatory control and articulatory function with DBS, other findings highlight DBS-induced dysarthria as a frequent adverse effect [13–18].

Given these inconsistencies, further research with robust methodology and sufficient sample size is warranted. The present study aims to evaluate the impact of STN-DBS on voice, acoustic parameters and speech features in a large PD cohort, thereby contributing objective evidence to the existing literature.

## Materials and methods

### Study design and participants

This study was conducted between August 2023 and September 2025 between the Departments of Otorhinolaryngology, Neurosurgery, and Neurology at Basaksehir Cam and Sakura City Hospital, University of Health Science. It was designed as an analytical, observational cohort study and approved by the Basaksehir Cam and Sakura City Hospital Institutional Ethics Committee (Approval No: 2024-KAEK-11).

Patients indicated for STN-DBS due to motor symptom progression underwent preoperative evaluation one day prior to surgery, including videolaryngostroboscopy. Voice recordings were collected only from individuals with normal laryngeal, oral cavity, and vocal fold examination findings. Postoperatively, following DBS activation and stabilization of voltage parameters, voice recordings were repeated at the third month and compared with baseline measurements.

Exclusion criteria included irregular follow-up, active upper respiratory tract infection, comorbid conditions affecting speech or respiration (e.g., vocal fold lesions, obstructive/restrictive lung disease), velopharyngeal insufficiency, and prior oropharyngeal surgery.

During both pre- and postoperative assessments, the following variables were recorded: age, sex, PD subtype (Tremor-Dominant [TD] or Akinetic-Rigid [AR]), UPDRS-III motor scores (medication-on state), and stimulation parameters including right/left STN voltage (mA), pulse width (µs), and frequency (Hz). All patients underwent UPDRS-III scoring and comprehensive voice-speech evaluation prior to surgery [19].

Postoperatively, patients were followed by a movement-disorders neurologist. Levodopa doses were reduced by approximately 30–35% during the first three months to minimize medication-related confounding on speech outcomes. The third postoperative month was selected as the evaluation time point to ensure stabilization of stimulation effects while limiting disease-related progression.

### Surgical procedure

All patients underwent standard stereotactic DBS surgery. One day prior to the operation, magnetic resonance imaging (MRI) was performed to directly target the subthalamic nucleus (STN). On the morning of surgery, an Integra^®^ CRW^®^ stereotactic frame was applied, followed by a 1-mm slice-thickness computed tomography (CT) scan. CT images were fused with the MRI scans for surgical planning, and all image processing was performed using BrainLab Elements^®^.

The procedure was carried out in two stages. In the first stage, leads were placed bilaterally on the STN under local anesthesia using Boston Scientific’s Vercise Cartesia™ Directional Lead.Before final lead placement, microelectrode recording (MER) and intraoperative test stimulation were performed to verify the optimal target site. In the second stage, performed under general anesthesia on the same day, the pulse generator (Boston Scientific Vercise Genus™ P-R16) was implanted in the left subclavian region. A postoperative CT scan with 1-mm slice thickness, oriented perpendicular to the lead axis, was obtained at the sixth postoperative hour to radiologically confirm the accuracy of the target localization.

### Acoustic analysis

Acoustic parameters of voice were analyzed using Dr. Speech software (Tiger Rehabilitation Medical Technology Co., Ltd., Shanghai, China), which provides numerical data for fundamental frequency, intensity, and related parameters. Recordings were obtained at a sampling rate of 44,100 Hz using a Fenix USB-280 USB Condenser Microphone Setpositioned approximately 5 cm from the mouth. Participants were instructed to phonate the vowel /a/ at their habitual speaking pitch and loudness for 5 s.

The following parameters were measured: fundamental frequency (F0), standard deviation of F0 (SD F0), maximum and minimum F0 values (max. F0, min. F0), jitter (Jitt, %), shimmer (Shim, %), and harmonic-to-noise ratio (HNR). Subsequently, the maximum phonation time (MPT) was determined using the longest sustained /a/ phonation achievable after a single deep inhalation. The MPT measurement was repeated three times, and the longest duration was recorded for analysis. Thus, sustained vowel phonation was used exclusively for instrumental acoustic analysis, whereas connected speech tasks were employed for intelligibility and discourse-level evaluation (see Sect.  2.4.3).

### Auditory–perceptual evaluation of voice

#### GRBAS scale

The perceptual evaluation of the recorded voice samples was conducted using the GRBAS scale [20] by three independent speech and language pathologists. The GRBAS scale assesses five perceptual dimensions of voice—Grade (overall severity of dysphonia), Roughness, Breathiness, Asthenia (weakness), and Strain—each rated subjectively on a four-point scale ranging from 0 to 3, where 0 = normal, 1 = slight abnormality, 2 = moderate abnormality, and 3 = severe abnormality. The degree of voice disorder was determined separately for each parameter by assigning a score between 0 and 3. For each participant, the scores of all five parameters were recorded separately for male and female groups. To minimize inter-rater variability and enhance objectivity, all recordings were re-evaluated one week later in a randomized order, and new scores were assigned. The mean of the three raters’ scores for each parameter was calculated, and preoperative and postoperative results were compared accordingly.

#### Voice handicap index-10 (VHI-10)

As one of the perceptual evaluation tools, the Turkish version of the Voice Handicap Index-10 (VHI-10) whose validity and reliability have been previously established—was utilized in this study [21]. The VHI-10 is a self-administered questionnaire consisting of 10 items, each rated on a five-point Likert scale from 0 to 4. Higher total scores indicate a greater perceived severity of voice-related difficulties. The index has been adapted into multiple languages beyond English, with validation and reliability studies conducted for each version [21, 22]. Rather than differentiating between specific pathologies, the primary purpose of the VHI-10 is to capture the individual’s subjective perception of their own voice problems. In this study, patients’ preoperative and postoperative VHI-10 scores were recorded, and mean values were calculated and compared separately for male and female participants.

#### Evaluation of accuracy and intelligibility in verbal production

To capture functional, connected speech rather than isolated phonation, speech intelligibility was assessed using recorded speech samples obtained during standardized text reading and narrative/discourse tasks. Participants in the study group were instructed to read the standardized Turkish passage “Atlıkarınca” the Turkish equivalent of the Rainbow Passage which contains all Turkish phonemes in various phonetic positions. In addition, narrative samples were recorded as patients described picture sequences taken from the story description section of the Turkish Aphasia Test [23]. Spontaneous speech samples were also collected to reflect natural speech patterns.

From each patient’s recordings, segments of at least 200 words were selected, and the number of correctly produced words was determined to calculate the percentage of correct words. To minimize inter-rater variability and ensure objective evaluation, all recordings were re-listened one week later in randomized order and rescored. The evaluations were conducted independently by three blinded certified speech and language pathologists, and the final intelligibility score for each participant was obtained by averaging the percentages of correctly produced words calculated by the three raters.

### Statistical analysis

Sample size estimation was performed using GPower software, version 3.1.9.6 [24]. The minimum required sample size was calculated based on an alpha error of 5%, a statistical power of 90%, and an effect size of 0.4. Data analysis was conducted using the Jamovi software (The Jamovi Project, 2024, v2.6) and the R statistical programming language (R Core Team, 2024, v4.4) [25, 26]. Descriptive statistics for continuous variables were presented as mean, standard deviation, minimum, and maximum values, while categorical variables were summarized as frequency (n) and percentage (%). The Kolmogorov–Smirnov test was used to assess the normality of data distribution. For dependent variables not conforming to normal distribution, nonparametric Wilcoxon signed-rank tests were applied. Statistical significance was set at *p* < 0.05.

## Results

### Participant characteristics

A total of 111 patients who were evaluated and indicated for STN-DBS by a multidisciplinary movement disorders surgery council—comprising neurologists, neurosurgeons, psychiatrists, otorhinolaryngologists, speech and language pathologists, and psychologists—were enrolled in the study. Four patients who did not attend follow-up visits (and thus did not complete the second phase of the study) and two patients who spoke a foreign language and could not be adequately assessed were excluded. Consequently, statistical analyses were conducted on data from 105 patients. Of these, 55 were female (52.4%) and 50 were male (47.6%). The mean age was 57.3 years (range: 35–71) for females and 57.7 years (range: 23–74) for males.

Regarding subtype distribution, 73 patients (69.5%) were classified as having the akinetic-rigid (AR) phenotype, while 32 patients (30.5%) exhibited the tremor-dominant (TD) phenotype. Preoperatively, the mean UPDRS-III motor score in the medication-on state was 19.4, whereas at postoperative follow-up visits, the mean score decreased to 13.6 (Table 1). In the postoperative period, improvement in UPDRS-III scores was observed in 77 patients (73.3%), while 28 patients (26.7%) demonstrated worsening (Table 1).

**Table 1:** Demographic and clinical characteristics of the participant

		Total (*n* = 105)	Female (*n* = 55)	Male (*n* = 50)
Mean Age		57.5 ± 9.03	57.3 ± 8.57	57.7 ± 9.59
Disease Duration		11.5 ± 6.08	11.5 ± 5.1	11.6 ± 7.06
UPDRS3	Preoperative	19.4 ± 9.16	19.3 ± 8.53	19.5 ± 9.9
	Postoperative 3. month	13.6 ± 11.7	14.4 ± 11.6	12.6 ± 11.8
Electrode targetting		STN	STN	STN
Laterality		Bilateral	Bilateral	Bilateral
Lead placement confirmation		Postoperative 6.hour CT	Postoperative 6.hour CT	Postoperative 6.hour CT
Voltage (mA)	Right	0.79 ± 0.30	0.80 ± 0.28	0.79 ± 0.31
	Left	0.49 ± 0.22	0.49 ± 0.23	0.48 ± 0.21
Pulse Width (µs)	Right (30–70)	60.3	60.5	60
	Left (30–70)	60.3	60.5	60
Frequency (Hz)	Right (130–179)	141 ± 20.3	140 ± 19.6	142 ± 21.1
	Left (120–179)	141 ± 20.3	140 ± 19.6	142 ± 21.1
Type of Parkinson’s Disease	Akinetic-Rigid (AR)	73 (%69.5)	40 (%38.1)	33 (%31.4)
	Tremor-Dominant (TD)	32 (%30.5)	15 (%14.3)	17 (%16.2)

STN: Subthalamic nucleus, CT: Computer tomography

The mean duration of disease among patients was 11.5 years. Demographic characteristics and related findings of the participants are presented in Table 1 (*p* < 0.05). As shown in Table 1, the mean voltage values of the right electrode (lead) were higher compared to the left side. For the left STN, the mean stimulation parameters were 0.49 mA (range: 0.3–2.9 mA), 59.5 µs (range: 30–70 µs), and 142.5 Hz (range: 120–179 Hz). For the right STN, the corresponding values were 0.79 mA (range: 0.3–2.9 mA), 60 µs (range: 30–70 µs), and 143.1 Hz (range: 130–179 Hz).

### Objective evaluation methods

#### Acoustic analysis results

The findings of the acoustic analysis are summarized in Table 2. When comparing the pre- and postoperative results, no statistically significant differences were observed in fundamental frequency, shimmer, harmonic-to-noise ratio, or maximum phonation time for either female or male patients. (*p* > 0.05) The numerical values before and after the procedure exhibited similar characteristics overall. However, an improvement in jitter values was observed in both groups, and this change was found to be statistically significant. (*p* < 0.05)

**Table 2:** Acoustic analysis results of the participants

	Total (*n* = 105)			Female (*n* = 55)			Male (*n* = 50)		
	Preoperative	Postoperative 3rd month	*p* value	Preoperative	Postoperative 3rd month	*p* value	Preoperative	Postoperative 3rd month	*p* value
F0 (Hz)	173 ± 45.6	177 ± 51.6	0.210	201 ± 40	204 ± 51.9	0.716	141 ± 27.3	146 ± 30.5	0.267
SD F0 (Hz)	4.37 ± 4.7	3.55 ± 2.79	0.107	4.85 ± 5.56	3.82 ± 1.79	0.183	3.83 ± 3.52	3.26 ± 3.59	0.371
F0 min. (Hz)	161 ± 45	165 ± 45	0.128	188 ± 40.6	190 ± 41.4	0.492	131 ± 27.4	137 ± 29.8	0.143
F0 max. (Hz)	188 ± 50.3	190 ± 57	0.699	219 ± 46	216 ± 53.4	0.649	155 ± 29.4	160 ± 45.7	0.317
Jitter (%)	0.39 ± 0.33	0.28 ± 0.25	0.008*	0.34 ± 0.32	0.24 ± 0.23	0.007*	0.43 ± 0.35	0.33 ± 0.36	0.005*
Shimmer (%)	2.8 ± 1.73	2.55 ± 1.88	0.140	2.55 ± 1.65	2.18 ± 1.66	0.055	3.07 ± 1.70	2.96 ± 1.22	0.318
HNR (dB)	22.9 ± 4.73	23.5 ± 5.72	0.230	23.7 ± 4.32	23.8 ± 5.0	0.818	22 ± 4.21	23.2 ± 4.41	0.122
MPT (sn)	10.9 ± 5.49	11.1 ± 5.94	0.804	10 ± 4.9	10.4 ± 3.2	0.569	11.9 ± 5.0	11.8 ± 5.6	0.877

*F0* Fundamental Frequency, *HNR* Harmonics-to-noise ratio, *MPT* Maximum phonation time

### Auditory–perceptual evaluation results

#### GRBAS

**Fig. 1:**
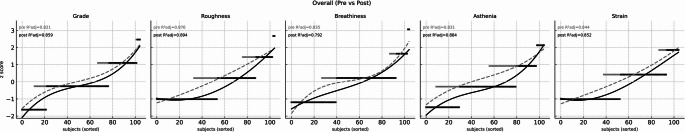
Overall Pre- and Post-treatment fitted-curve analysis showing changes in GRBAS z-score distributions. Each panel displays the sorted z-score distributions for pre-treatment (dashed fitted line) and post-treatment (solid fitted line) GRBAS scores. Z-scores were calculated using pooled pre- and post-treatment values to enable direct visual comparison. Adjusted R² values represent the model fit for the respective polynomial trend lines

**Fig. 2:**
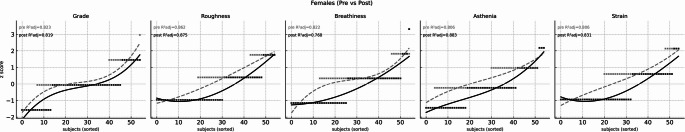
Pre- and post-treatment fitted-curve comparison of GRBAS parameters in female participants. Sorted z-score distributions are shown for pre-treatment (dashed line) and post-treatment (solid line) scores among females. Post-treatment curves exhibit a consistent downward displacement relative to pre-treatment curves, reflecting perceptual improvement across all GRBAS domains. Adjusted R² values indicate adequate fit of the polynomial trend lines within this subgroup

**Fig. 3:**
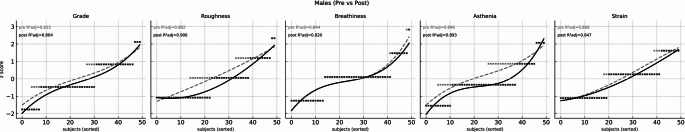
Pre- and post-treatment fitted-curve comparison of GRBAS parameters in in male participants. Each panel presents sorted z-score distributions for pre-treatment (dashed line) and post-treatment (solid line) GRBAS ratings among male participants. Similar to females, males demonstrate a downward shift in fitted curves following treatment, indicating overall improvement. Adjusted R² values denote the fit strength of each polynomial model

The GRBAS perceptual evaluation results are presented in Table 3. In the female PD group, all five GRBAS parameters showed statistically significant postoperative improvement (*p* < 0.05). In male patients, Grade, Roughness and Asthenia improved significantly (*p* < 0.05), while Breathiness and Strain demonstrated a non-significant tendency toward improvement (*p* > 0.05). Overall, perceptual analysis suggested a favorable postoperative shift across parameters. Pre–post fitted-curve plots illustrated a consistent downward displacement in severity across all GRBAS dimensions, indicating reduced perceptual burden and improved vocal quality. Postoperative z-score dispersion also decreased, reflecting greater vocal homogeneity, whereas preoperative distributions showed wider variability and clustering at higher severity levels. These patterns suggest improvements were group-wide rather than limited to isolated patients (Figs. 1, 2 and 3). Although all dimensions improved, the magnitude differed by parameter: Grade and Roughness exhibited the most pronounced divergence, while Asthenia showed the largest mean change and strongest effect size. Breathiness and Strain improved more modestly but still showed smoother postoperative trajectories with fewer high-severity cases, highlighting the multidimensional and parameter-specific nature of recovery. Sex-stratified analyses confirmed broadly similar improvement patterns, with males displaying slightly greater baseline variability in Grade and Roughness; however, postoperative change direction was consistent across sexes (Figs. 2 and 3).

**Table 3:** Auditory-perceptual evaluation (GRBAS Scale) results of the participants

	Total (*n* = 105)			Female (*n* = 55)			Male (*n* = 50)		
	Preoperative	Postoperative 3rd month	*p* value	Preoperative	Postoperative 3rd month	*p* value	Preoperative	Postoperative 3rd month	*p* value
Grade	1.3 ± 0.7	1.09 ± 0.74	< 0.001*	1.16 ± 0.66	0.90 ± 0.64	0.003*	1.44 ± 0.73	1.28 ± 0.80	0.050*
Roughness	0.98 ± 0.8	0.66 ± 0.79	< 0.001*	0.87 ± 0.74	0.54 ± 0.68	< 0.001*	1.10 ± 0.86	0.80 ± 0.88	< 0.001*
Breathiness	0.93 ± 0.69	0.75 ± 0.7	0.006*	0.92 ± 0.63	0.61 ± 0.68	< 0.001*	0.94 ± 0.76	0.90 ± 0.7	0.694
Asthenia	1.45 ± 0.74	1.02 ± 0.85	< 0.001*	1.44 ± 0.71	0.94 ± 0.87	< 0.001*	1.46 ± 0.78	1.10 ± 0.83	< 0.001*
Strain	0.81 ± 0.7	0.60 ± 0.67	< 0.001*	0.76 ± 0.66	0.45 ± 0.6	< 0.001*	0.86 ± 0.75	0.76 ± 0.71	0.261

Statistical significance was set at *p* < 0.05.

#### Voice handicap index

The results of the Voice Handicap Index (VHI) are summarized in Table 4. When evaluated regardless of gender, improvement was observed in 86 patients (81.9%), while deterioration was noted in 19 patients (18.1%). For the entire patient cohort, the mean preoperative VHI score was 22.7, whereas the postoperative mean score was 21.8, and this difference was statistically significant. (*p* = 0.031)

**Table 4:** Auditory-Perceptual evaluation results (VHI and PCW Scales) of the participants

	Total (*n* = 105)			Female (*n* = 55)			Male (*n* = 50)		
	Preoperative	Postoperative 3rd month	*p* value	Preoperative	Postoperative 3rd month	*p* value	Preoperative	Postoperative 3rd month	*p* value
VHI-10	22.7 ± 7.92	21.8 ± 7.64	0.031*	27.5 ± 6.8	25.9 ± 5.9	0.004*	17.5 ± 7.1	17.3 ± 6.5	0.763
PCW	70.1 ± 13.8	71.7 ± 14.7	0.014*	62.8 ± 12.1	65.1 ± 11.2	0.020*	78.1 ± 13.1	78.9 ± 12.2	0.332

*PCW* Percentage of correct words, *VHI* Voice handicap index, Statistical significance was set at *p* < 0.05

In the female patient group, improvement was detected in 47 patients (85.5%), while 8 patients (14.5%) showed deterioration. The mean preoperative score was 27.5, and the postoperative score was 25.9; this change was statistically significant. (*p* = 0.004)

In the male patient group, improvement was observed in 39 patients (78%), and deterioration in 11 patients (22%). The mean preoperative score was 17.5, and the postoperative score was 17.3; this difference was not statistically significant. (*p* = 0.763)

#### Accuracy and intelligibility evaluation

The intelligibility evaluation results are summarized in Table 4. Assessments were conducted based on the mean percentage of correctly produced words, calculated independently by three speech and language therapists. When all patients were evaluated together, an increase was observed in 69 patients (65.7%), while a decrease was noted in 36 patients (34.3%). The mean percentage of correctly produced words increased from 70.1% before the procedure to 71.7% after the procedure, and this change was statistically significant (*p* = 0.01).

In the female patient group, the mean percentage of correctly produced words increased from 62.8% preoperatively to 65.1% postoperatively, with this difference being statistically significant (*p* = 0.02). Improvement in intelligibility was observed in 38 female patients (69.1%), whereas deterioration was observed in 17 patients (30.9%). In the male patient group, the mean percentage of correctly produced words was 78.1% before and 78.9% after the procedure; however, this change was not statistically significant (*p* = 0.33). Improvement was observed in 31 male patients (62%), while deterioration was noted in 19 patients (38%). Group level intelligibility outcomes showed a significant improvement in the overall cohort (*p* = 0.01), with 69.1% of females and 62% of males demonstrating postoperative gains, while the remaining participants exhibited varying degrees of decline.

When compared with clinical and demographic findings, word accuracy and intelligibility were found to decline particularly in older patients and in those with higher UPDRS-III scores. In contrast, no relationship was identified between intelligibility measures and variables such as sex, Parkinson’s disease subtype, stimulation parameters, or speech and acoustic characteristics.

## Discussion

This study represents one of the most comprehensive investigations to examine the effects of subthalamic deep brain stimulation (STN-DBS) on acoustic parameters and speech features. We prospectively evaluated acoustic outcomes, perceptual voice measures, and speech intelligibility in patients undergoing STN-DBS. Importantly, the speech protocol incorporated sustained vowel phonation for acoustic analysis and connected-speech tasks (standardized passage reading, picture-based narrative, and spontaneous speech) for intelligibility assessment, ensuring evaluation beyond isolated phonatory performance.

The literature generally supports the motor benefits of STN-DBS, yet its impact on speech remains inconsistent. Reports of postoperative speech deterioration range between 9 and 69% [27–29], whereas other studies have demonstrated improvements in selected parameters [12, 30, 31]. Acoustic analysis serves as an objective tool for evaluating voice characteristics such as fundamental frequency, perturbation metrics, and vocal stability. While some studies reported postoperative improvements in acoustic measures [13–15], others documented deterioration [18, 32, 33]. In our cohort, acoustic parameters did not differ significantly pre- vs. post-operatively in either sex, except for a significant reduction in jitter among female patients, suggesting that STN-DBS did not produce robust negative or positive acoustic effects.

Roman et al. similarly reported no significant change in fundamental frequency or perturbation indices following DBS [17], whereas Saraç et al. observed a trend toward deterioration, particularly in female patients, without corresponding GRBAS worsening [34]. Our findings instead revealed perceptual improvements: all GRBAS dimensions improved significantly in females, and Grade, Roughness, and Asthenia improved significantly in males, with Breathiness and Strain showing non-significant positive trends. These results indicate that despite limited acoustic change, perceptual voice quality may improve after STN-DBS. Previous work by Tripoliti et al. noted a 50% improvement in motor function accompanied by a 50% reduction in intelligibility [28]. In contrast, our study showed improved intelligibility in 69.1% of females and 62% of males.

In our study, speech intelligibility was derived from blinded transcription accuracy of connected speech samples, which is a robust method for observing real-world changes. In our cohort, VHI-10 scores showed a statistically significant decrease (22.7 to 21.8), and speech intelligibility showed a modest increase (70.1% to 71.7%). While these changes reached statistical significance, we must acknowledge that they fall below the established thresholds for Minimal Clinically Important Difference (MCID); for instance, the MCID for VHI is typically considered to be a change of at least 5 points. Therefore, rather than a robust functional improvement, these results are better interpreted as a demonstration of functional stability in voice and speech following STN-DBS. Given the progressive nature of Parkinson’s disease, maintaining speech functions and preventing the expected decline is a clinically valuable outcome. Furthermore, as suggested by the concurrent improvement in perceptual GRBAS ratings and intelligibility in some subjects, the combined effect of these modest gains might lead to a more detectable auditory benefit for some listeners than any single metric would suggest.

Recent studies have suggested that the characteristics of STN-DBS and the stimulation parameters used (voltage, pulse width, and frequency) may influence voice quality [14, 18]. In a recent study by Bobin et al., it was reported that stimulation of specific subregions of the subthalamic area at particular voltage intensities is the main determinant of speech-related changes. Traditionally, speech alterations have often been overlooked, with the improvement of motor function being considered the primary objective of DBS. However, the authors emphasized that carefully adjusted stimulation regimens, taking into account central stimulation characteristics, could achieve improvements in both motor and speech outcomes [12].

In our study, electrode placement was performed in the posterolateral STN region, which has been associated with more favorable speech outcomes in recent literature [12, 35]. This standardization likely minimized implantation-site variability. Additionally, previous research suggests that right-sided stimulation may exert more positive speech effects compared with left-sided stimulation [28, 36], a finding that aligns with our perceptual data. As reflected in Table 1, higher voltage values were applied to the right STN, which may have contributed to improved speech parameters.

Although some studies have indicated that bilateral stimulation yields superior outcomes [37, 38], we were unable to isolate laterality-specific effects because all patients received bilateral DBS. Aldridge et al. reported that stimulation amplitudes ≥ 3.5 V may negatively affect acoustic features and speech performance [39]; however, our stimulation range did not exceed this threshold, and our short-term design prevents conclusions about potential high-voltage effects.

A recent meta-analysis highlighted that variability in speech response to DBS arises from complex interactions among neural circuitry, individual anatomy, stimulation spread, cognitive features, and personal factors [36]. Understanding how these elements shape speech outcomes may allow for optimization of lateralization, contact selection, and stimulation parameters to preserve or improve communication. In the context of this evidence, our findings further support the multifactorial nature of DBS effects on speech.

From an otolaryngology and phoniatrics perspective, these findings hold clinical significance. Although DBS is neurosurgical in origin, its outcomes directly affect laryngeal physiology, voice quality, respiratory-phonatory coordination, and communicative intelligibility core domains of ENT and speech science practice. Structured pre- and postoperative voice assessment should therefore be considered within DBS management.

ENT and speech specialists play a critical role in identifying DBS-related dysphonia, monitoring laryngeal biomechanics, initiating voice therapy when needed, and collaborating with neurology to modify stimulation parameters when speech decline occurs. Our observations regarding stimulation laterality and perceptual-acoustic patterns underscore the importance of interdisciplinary care in preserving communication outcomes. Integrating routine voice assessment, acoustic monitoring, and intelligibility testing into DBS follow-up protocols may enable early detection of adverse vocal effects and facilitate individualized programming strategies.

In our study, to minimize potential confounding factors, all patients underwent posterolateral electrode placement, dopaminergic medication doses were uniformly reduced by approximately 30–35%, and bilateral stimulation was applied. We believe that this methodological standardization enhanced the reliability of our results. To the best of our knowledge, our research represents one of the few studies in the literature that evaluates acoustic analysis and speech parameters in such detail and within a large patient population. With the reliable data obtained, this study provides a significant contribution to the scientific literature.

This study has limitations. First, the design focused on short-term outcomes. Although this limitation was an intentional design choice, longer follow-up is necessary to capture delayed neuromodulatory changes and distinguish treatment-related effects from natural disease progression. Second, the absence of a medical-management-only control group prevents definitive attribution of speech changes solely to DBS. Without a pharmacological-only comparator, improvements or deterioration may reflect medication adjustments, progression, or individual variability. Future trials incorporating a medical-only control cohort will clarify treatment-specific effects. Although the total sample size satisfied the a priori power calculation for primary analysis (α = 0.05, power = 90%, effect size = 0.4), subgroup analyses (sex, age, stimulation side) remain underpowered and should be interpreted cautiously. Additionally, the relatively low stimulation voltage levels used in this study may have restricted the observation of potential speech and voice related effects associated with higher stimulation parameters. Multi-center studies with larger cohorts, longitudinal monitoring, and variable stimulation protocols are warranted to verify subgroup trends and refine speech outcome prediction.

## Conclusion

This study, characterized by a large patient population and methodological standardization including posterolateral electrode placement, uniform reduction of dopaminergic medication doses, and the use of bilateral stimulation demonstrates that STN-DBS provides significant improvement in motor symptoms while exerting complex and multifactorial effects on speech and acoustic parameters. Although no significant changes were observed in acoustic analyses, improvements were detected in GRBAS scores and intelligibility parameters, particularly among female patients. Our findings stand out by contrasting with previous reports describing adverse speech outcomes and highlight the multifactorial nature of DBS effects on speech. Therefore, future multicenter studies with long-term follow-up are warranted to clarify the uncertainties surrounding speech functions and to provide more reliable contributions to the existing literature.

## Data Availability

Data supporting of the this study results are available from Ozan Tuysuz upon reasonable request.
